# An eUtils toolset and its use for creating a pipeline to link genomics and proteomics analyses to domain-specific biomedical literature

**DOI:** 10.1186/2043-9113-2-9

**Published:** 2012-04-16

**Authors:** Prakash M Nadkarni, Chirag R Parikh

**Affiliations:** 1 Department of Medicine, Program of Applied Translational Research, Yale University School of Medicine, New Haven, CT, USA; 2 Clinical Epidemiology Research Center, Veterans Affairs Medical Center, West Haven, CT, USA

**Keywords:** Entrez Programming Utilities, Proteomics Analysis, Pubmed filters

## Abstract

**Background:**

Numerous biomedical software applications access databases maintained by the US National Center for Biotechnology Information (NCBI). To ease software automation, NCBI provides a powerful but complex Web-service-based programming interface, eUtils. This paper describes a toolset that simplifies eUtils use through a graphical front-end that can be used by non-programmers to construct data-extraction pipelines. The front-end relies on a code library that provides high-level wrappers around eUtils functions, and which is distributed as open-source, allowing customization and enhancement by individuals with programming skills.

**Methods:**

We initially created an application that queried eUtils to retrieve nephrology-specific biomedical literature citations for a user-definable set of genes. We later augmented the application code to create a general-purpose library that accesses eUtils capability as individual functions that could be combined into user-defined pipelines.

**Results:**

The toolset’s use is illustrated with an application that serves as a front-end to the library and can be used by non-programmers to construct user-defined pipelines. The operation of the library is illustrated for the literature-surveillance application, which serves as a case-study. An overview of the library is also provided.

**Conclusions:**

The library simplifies use of the eUtils service by operating at a higher level, and also transparently addresses robustness issues that would need to be individually implemented otherwise, such as error recovery and prevention of overloading of the eUtils service.

## Background

### Motivation for the present work

An ancillary goal of the TRIBE-AKI consortium (Translational Research Investigating Biomarker Endpoints in Acute Kidney Injury, http://www.yale.edu/tribeaki/), supported by the US National Institutes of Health, is to identify novel biochemical markers that may be sensitive, early-onset, or etiology-specific indicators of acute renal injury. One approach is to look for proteomics-based markers that are differentially expressed in patients with renal injury of known etiology, and then identify, through a biomedical literature search, hypotheses that link the involved proteins (or the underlying genes) to renal disease, in order to suggest further confirmatory experiments. Because the number of statistically significant signals tends to be very large, partial automation of this task is desirable.

The National Center for Biotechnology Information (NCBI) maintains numerous databases (Genbank, Pubmed, Protein, etc.,) that are essential tools for the life science community. It also provides a programming interface, the Entrez Programming Utilities (eUtils) [1], available via a Web Service, which allows almost complete automation of operations that would otherwise be performed manually. While eUtils is elegantly designed and powerful, there are numerous complexities involved in building applications that make use of eUtils, as discussed shortly in Overview of eUtils. Therefore it is desirable to create a higher-level interface that simplifies its use.

This manuscript describes a toolset that allows such simplification. The toolset includes a code library can be integrated into custom applications that need to access eUtils, as well as a user interface that can be operated interactively to retrieve data sets in a series of steps. We illustrate its use with an application that performs domain-specific literature surveillance related to a continually expanding set of markers of biological interest.

### Previous related work

Gene-expression and proteomics experiments are valuable high-throughput techniques for exploring disease mechanisms [2], and are complemented by retrieval of the literature and ancillary information (e.g., MEDLINE keywords) related to differentially expressed genes or proteins. Below, we broadly categorize the purposes of literature-retrieval efforts, and identify previous work in each category.

1. Automated Annotation: PubMatrix [3], is used to query PubMed using two term lists of keywords terms, and outputs a matrix recording the co-occurrence counts for any term-pair (along with the citations in each matrix cell). This information can be used to annotate gene clusters. CoPub[4] takes a gene list and, using Unified Medical Language System [5] co-occurrence data, returns keywords that co-occur with the gene names in the literature. This information is now used to retrieve the PubMed abstracts where the co-occurrences were found. MILANO [6] uses a similar approach, but allows the terms to be user-defined, and also implements query expansion by gathering all gene-name synonyms.

2. Dimension reduction: It is desirable to compress the very numerous positive individual-gene signals into a succinct, biologically meaningful interpretation. One standard summarization approach is to relate the signals to a smaller set of biological pathways or functions, using concept hierarchies such as Gene Ontology (GO) [7]. GO, however, is a continual work-in-progress whose utility, in turn, is based on ongoing curatorial surveillance of published research: the variability of research activity in different areas leads to variation in comprehensiveness of different parts of the GO hierarchy. Consequently, experimenters often wish to go directly to the source literature, using alternative dimension-reduction methods.Thus, Jenssen et al[8], by text-mining MEDLINE titles and abstracts, created a gene-to-gene “co-citation network”. PubGene, with co-citations annotated with GO and Medical Subject Heading (MeSH) terms. The work of Masys et al [9] uses shared MeSH terms (as well as hierarchical relationships between such terms) to automatically cluster gene-expression results, while Bressel et al [10] display links between genes (based on MeSH and GO terms) using a tree structure.

3. Literature Surveillance and filtering of results to areas of specific research interest. GenDrux[11], queries PubMed to identify disease-gene-drug relationships; the pilot implementation has been applied to the breast cancer area. The present paper’s work falls into this category.

### Experimental context

#### Use of domain-specific medline content filters

The papers of Garg et al and [12] and Iansavichus et al [13] describe the creation of Medline filters to allow clinicians to search for articles within a specific clinical discipline: they have devised two filters with high sensitivity and specificity respectively for the nephrology domain. These filters (specified using Pubmed query syntax [14]) are:

High specificity: “(renal replacement therapy[majr] OR kidney diseases[majr] OR kidney[ti] OR nephr*[ti] OR renal[ti] OR kidney[majr:noexp] OR renal dialysis[mh] OR kidney function tests[majr] OR proteinuria[majr:noexp] OR glomerul*[ti]) NOT (kidney neoplasms[majr] OR pyelonephritis[majr:noexp] OR urinary tract infections[majr] OR nephrolithiasis[majr])”High-Sensitivity: “kidney diseases[mh] OR renal replacement therapy[mh] OR renal[tw] OR kidney*[tw] OR (nephre*[tw] OR nephri*[tw] OR nephroc*[tw] OR nephrog*[tw] OR nephrol*[tw] OR nephron*[tw] OR nephrop*[tw] OR nephros*[tw] OR nephrot*[tw]) OR proteinuria[tw]”

Such filters can be developed for any area of interest: evaluating a candidate filter, however, is a painstaking process. It requires initially identifying a set of target documents that domain experts have determined are relevant, and then running the candidate filter and determining how many of the target documents appear in the result set, and in what proportion. A sensitive filter will maximize the proportion of target documents, while a specific filter, while possibly retrieving fewer documents, will minimize the number of non-target documents that are considered non-relevant.

#### Overview of eUtils

eUtils provides access to the individual Entrez databases as well as to the cross-links between them. Its functionality can be broadly divided into the following categories:

1. *Executing complex Boolean or keyword-based searches* of individual databases using Entrez query syntax. The results are sets of unique IDs corresponding to objects from that database (e.g., PMIDs for PubMed citations, GenInfo IDs for Genbank sequences, etc.). This may be augmented by a “post” operation: saving a set of IDs for future use with subsequent search.The search can be modified to act as a *filtering operation*: that is, if one supplies a set of IDs and an query expression, the latter can be applied to the former to reduce the set (e.g., given a set of genes and the filter “Homo sapiens [organism]”, the genes are filtered to those in humans only. Similarly, one can apply content filters, as above, to restrict a set of PubMed IDs obtained by some other mechanism.

2. *Cross-linking operations.* Given a source database, a set of IDs from that database, and a destination database, return the linked IDs (against each source ID) from the latter. Only a subset of the databases is cross-linked. An extension of this operation (for PubMed) is to retrieve LinkOut URLs for a set of PubMed IDs.

3. *Information operations:* Given a database and a set of IDs relating to that database, retrieve either “summary” or detail information on the corresponding objects. The difference between the two retrieval modes is that summary format (a misnomer because complete data can be retrieved) consists of attribute-value pairs (often nested) that are typically presented as XML, while the detail format consists of individual named fields and is more suitable for tabular presentation. Detail format (retrieved by the “eFetch” functions within eUtils) is available for only some (commonly used) Entrez databases: other databases, notably the PubChem family, are retrievable only in summary format.

4. *Miscellaneous operations:* Summary information on the contents of individual databases, spelling suggestions (for PubMed). These are infrequently used in practice.

Several operations can be combined sequentially, with the Web service being optionally directed to “remember” intermediate results as “Environment” variables, each identified through a unique alphanumeric key. The appropriate use of such variables can save considerable to-and-fro network traffic and local input/output operations by saving the developer the trouble of downloading and saving these results.

As stated earlier, there are several intricacies involved in programming eUtils, which can potentially result in highly repetitious code to perform housekeeping chores and ensure code robustness.

· Errors (e.g., time-outs) must be trapped; one must check for zero results both for a service call as a whole, as well as for individual IDs in a list for which links must be retrieved.

· It is critical not to overwhelm the service: the eUtils documentation recommends that all lists be kept to 500 items or less. Very long ID lists, which may sometimes arise as intermediate result-sets within a batch of operations when the “environment” mechanism is being used, may cause a time-out or silent failure with mysterious errors if one tries to use them directly in a subsequent step. For example, attempting to filter a large intermediate list of IDs by a Boolean criterion may expand the result set instead of shrinking it. One must deal with large lists by writing loops that slice such lists into chunks not exceeding 500 items, with a service call being made against each chunk. Similarly, for queries returning numerous results, it is preferable to download them in 500-item chunks. To deal with intermediate result-sets robustly, it is often desirable to save them to disk.

· Much mechanical input–output code must be written: in the case of chunking operations, each operation’s results must be written to disk, and sometimes previously saved results must be read into memory to be passed to eUtils.

EUtils programming is therefore involved enough that it is desirable to create a high-level wrapper library around the suite that takes care of the above chores in a manner transparent to the programmer, with the top-level functionality accessible via single-line parameterized subroutine calls. The present work involves creating such a library, which is made available as open-source with accompanying documentation.

## Methods

We first describe the workflow of data retrieval related to domain-specific literature surveillance, and then describe the design principles of the toolset.

### Workflow of domain-related gene-literature surveillance

The objective of the workflow is to retrieve domain-specific (here, nephrology-related) PubMed data associated with a set of positive experimental signals. This can be divided into the following subtasks:

a. Transforming the output of the proteomics experiment (differentially expressed proteins, identified as either Gene Symbols or Accession Numbers) into a query expression that will return Geninfo IDs. This is done by composing a complex Boolean query (“OR”-ing the individual query terms, which are prefixed by the name of the field to which they refer – for example, “NP_000598 [accession]”). The final expression is filtered further by organism (Homo sapiens).

b. Retrieving and saving detail information on the genes of interest.

c. Retrieving PubMed IDs that are cross-linked with these genes (the result list can get very large, because a particular gene may have hundreds of associated citations), saving the list temporarily

d. Applying the two separate filters of Use of domain-specific medline content filters to the list (high-sensitivity and high-specificity). The high-sensitivity result set is a superset of the high-specificity result set. Both sets are saved locally, in the format (gene-id, Pubmed-ID), where the gene ID repeats as many times as there are associated citations.

e. Getting a list of unique citations: the same citation may apply to multiple genes if, for example, the paper describes a screening experiment on a family or panel of genes.

f. Fetching essential details of these citations (author list, title, journal, year, abstract, linkout information).

g. Storing the retrieved data for future use.

### The code library /front-end: design principles

While the nephrology literature-surveillance application accessed only a few databases, feedback from users convinced that access to a much wider set of Entrez databases – potentially every one that is eUtils-accessible – would be required. It was therefore important not to artificially limit the scope of the package.

A graphical front-end was deemed necessary to avoid limiting the use of the package to programmers only. Our design was intended to adhere to the design philosophy of UNIX filters: that is, the output of one step can be the input of another step, but each invocation performs only a single, simple task. Therefore, the idea was to allow users to construct miniature pipelines to accomplish a task. For the front-end to be considered useful, it had to be capable of allowing the user to interactively execute steps a-f of the above workflow. (For reasons discussed shortly, step g was determined to be beyond the scope of the tool.)

We used Visual Basic .NET (version 2010), part of Microsoft Visual Studio 2010, for code development. This development environment has the advantage of querying a Web service such as eUtils and automatically creating classes and subroutine definitions related to the service, thereby facilitating both exploration and programming of the service.

## Results

### Operation of software

**Figure 1:**
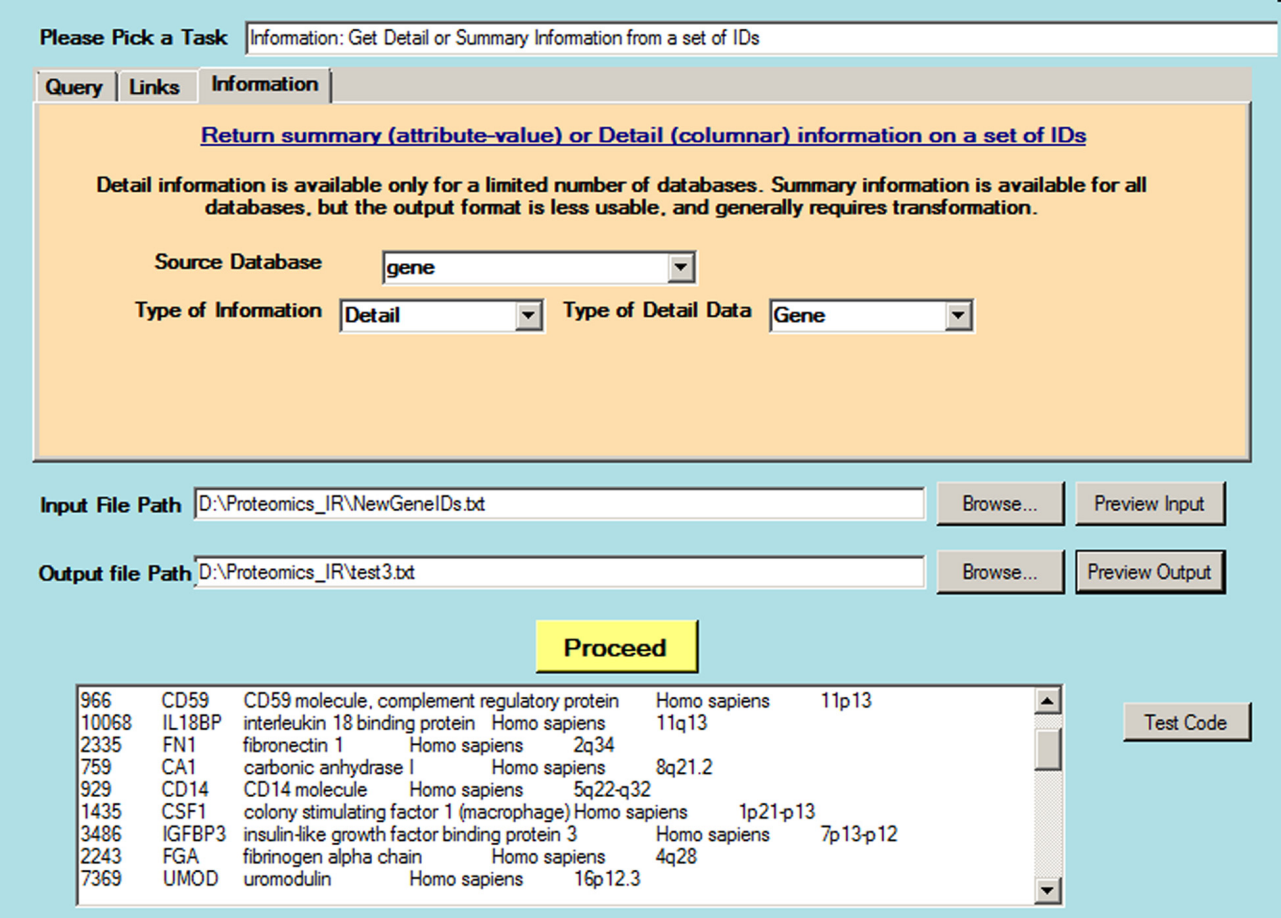
**A screen shot illustrating the citation database.** The background (sky blue) shows a gene (Agrin, human), and a list of citations in a table. The checkbox on the left of a given citation indicates that the citation is highly specific for nephrology, i.e., it has been retrieved using the “specific” Pubmed nephrology filter, which always retrieves a subset of the citations retrieved by the “sensitive” filter. Clicking the “Show Details of Selected Citation” button brings up the full details of the citation (including the Abstract) in an overlaid window (light cyan background).

The user interface is organized according to the categories of query/filter, links and information above, as shown in the screen-shot of Figure 1.

In the query interface, one either specifies as input set of identifiers in a text file containing identifiers, or specifies a query expression using Entrez syntax, which is most simply composed using the “Advanced Search” mechanism of Entrez itself and pasted into a text box. If a text file is used, it typically contains IDs, though gene accession numbers or gene symbols are also available for search options. The database must be specified from a pull-down, and the IDs must correspond to objects in the database – like Entrez itself, the front-end cannot verify that the specified IDs are valid entries for that database, other than by returning few or no results. Output is always written to a disk file that can be previewed, as shown in Figure 1: the input data can be similarly previewed.

Since most eUtils operations result in sets of IDs that can be used in subsequent operations, end-users can construct their own pipelines by using the output file of a given step as the input file of a subsequent step. Certain steps involve data reduction Thus, for step c, of Workflow of domain-related gene-literature surveillance, one must reduce the set of linked PubMed IDs to a unique set: the library therefore also includes a set of utility functions that extracts the contents of one or more columns from a delimited data file, optionally eliminating duplicate values, similar to the UNIX utility *uniq,* which gets unique values from a column of data).

Some elementary intelligence is built into the front-end, based on the eUtils documentation: the list of available databases varies according to the operation that the user chooses, and other options may also be restricted. Thus, for example, if the user chooses the Information operation and chooses the Structure database, only the “Summary” information format will be available.

In order to facilitate extraction of specific data from the datasets – such as step e in Workflow of domain-related gene-literature surveillance (getting unique values from a linked data-set) – the interface also includes extraction features that can extract one or more columns of data from a delimited text file, optionally creating unique values. For unique values, the user also has the option of sorting the values, generating sequential IDs (integers starting from 1), or generating frequency information (the number of times each value occurs). The last option is useful in certain circumstances: a citation that has a large frequency count (i.e., associated with numerous genes) is likely to be an “-omics” experiment. The sequential ID information is useful for string data that will be imported into a relational database, where every item has an associated unique identifier that serves as the primary key.

### Application to literature-surveillance

**Figure 2:**
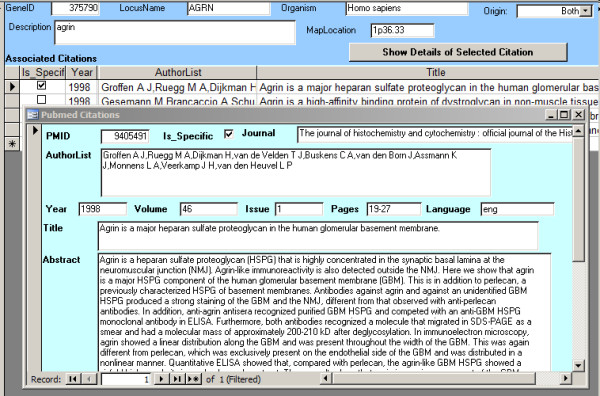
**A screen shot of the Entrez front-end application.** Details of a set of IDs previously downloaded from the NCBI databases (in this case, gene information from Genbank) are displayed as a preview in the lower window. Data is always downloaded as delimited text files, which are suitable for manipulation by a variety of programs, such as spreadsheets, as well as other parts of the front-end application: the preview is only intended for the user to glimpse the data to ensure that the data downloaded corresponds to what the user intended.

Steps a-f of the previously described workflow can be executed interactively. For data management (step g), locally saved data are bulk-imported into a relational database. Our local database application is currently in MS-Access format: as the data gets more voluminous, we expect to upsize this to Microsoft SQL Server, with the Access application serving as a front end. (Currently, the number of concurrent users is too few to justify the significant additional effort to create a Web front-end, but this situation may change.) Figure 2 shows a screen-shot of the database application, with an imported record.

Note that our tool reads/creates only tab-delimited files (output files have the column names as the first row): it is not intended to integrate closely with Microsoft Access. The reason for our decision is that different users prefer to use different data-management approaches (spreadsheets, Filemaker Pro, high-end DBMSs) and we did not wish to needlessly limit the tool’s use. (As an aid to the reader, we provide a reduced version of this database as Additional file 1).

## Discussion

### Limitations and future directions

One issue in creating any application that simplifies utilization of Entrez is how much functionality to reinvent. We have decided not to try to replicate the Advanced Search” Boolean query interface to the NCBI databases, since we are unlikely to improve on it. eUtils itself is continually enhanced (for example, detail information via the eFetch service family is periodically expanded to include additional databases), and we will have to update our library accordingly.

One drawback of the current design is that each step of a pipeline must be user-initiated through the graphical user interface. While convenient the first time, having to repeat the steps each time for tasks performed regularly with identical parameters or options can become tedious. Therefore it is desirable to create a simple macro-recording/playback facility to capture the user’s actions into a file that can be played back to execute these actions. As stated earlier, there are just four action categories – Query/Filter, Link, Extract and Information and therefore the actions can be represented in text using a straightforward syntax.

Such a facility could allow a standard user-devised sequence of operations to be run repeatedly. To be workable, the stored macro would have to use the syntax of a reasonably robust scripting language (so that we would not have to invent our own).

One scripting/automation language (for Windows) which we have used for reasonably complex tasks is AutoIt [15],whose syntax is modeled on Visual BASIC. While not open-source, AutoIT is freeware and supports interactive debugging: it is widely used by software testers for automated user-interface testing on the MS-Windows platform, because it can simulate keystrokes and mouse movements, and manipulate windows and processes to automate non-programmable software. We expect to have script generation ready for use in the next version of the software.

Based on demand, we may rewrite the library using Java so that it may run on the Macintosh and UNIX platforms.

## Conclusions

Our toolset simplifies use of the eUtils service by operating at a higher level, and also transparently addresses robustness issues that would need to be individually implemented otherwise, such as error recovery and prevention of overloading of the eUtils service. The graphical front-end makes most of this functionality available interactively to non-programmers. We hope that its free availability will make researchers and programmers who need to access NCBI content more productive.

### Availability and requirements

The application and documentation can be freely downloaded for use from the journal’s Website. (See Additional file 2). Its use is subject to the terms of the GNU General Public License v 3.0 [16].

## Competing interest

The authors declare that they have no competing interests.

## Authors’ contributions

Dr. Nadkarni developed the software. Dr. Parikh identified the research problem, provided the filter, and tested the output of the software. Both authors contributed to the writing of the manuscript.

## Supplementary Material

Additional file 1 This is a zipped Access database (Sample_Database.zip, which unzips to Sample_Database.mdb) that was used to produce the screenshot of Figure 1. It is intended to be used for illustration purposes only: its structure is specific to the current needs of the Parikh Lab, and its contents have been stripped down to a few records that illustrate its operation. Click here for file

Additional file 2 Software Bundle: This is a Zip file that contains a Microsoft Visual Studio Project, source code ( in Visual Basic.NET) , accompanying documentation in Microsoft Compiled HTML (.chm) format, as well as an executable for those who wish to use the program directly. The documentation includes a brief tutorial/manual for end-users in addition to descriptions of the library routines in MSDN (Microsoft Developer Network) format. After downloading and unzipping the contents on to your local desktop machine, the .chm file will not be immediately viewable on Windows Vista and Windows 7 (it is treated as “untrusted content”, so that, while you can see the table of contents, any attempt to view a topic will yield a message that “navigation to the Web page was cancelled”.). To make it viewable, right click on the file, choose “Properties” and then choose “Unblock” and “Apply”. Make sure that this file is on your *local* machine and not on a network drive: .chm file display over a network is blocked. If you wish to modify the source code, you will require some version of Microsoft Visual Studio 2008 or greater (Express Edition or greater) and will need to have .NET framework 3.5 or greater installed on your machine. This is pre-installed on Windows 7, and also available on XP and Vista through Windows update (Optional Updates). If you simply wish to run the application, make sure that the files EntrezAPI.pdb is in the same folder as EntrezAPI.exe: it contains configuration information for connecting to the eUtils Web service. Click here for file
